# Supplementary material for: Study of Concussion in Rugby Union through MicroRNAs (SCRUM): a study protocol of a prospective, observational cohort study
Source: BMJ Open. 2018 Nov 25;8(11):e024245. doi: 10.1136/bmjopen-2018-024245 (PMC6254405; doi:10.1136/bmjopen-2018-024245)
Supplement: Author's manuscript [file bmjopen-2018-024245.draft_revisions.pdf]

BMJ Open is committed to open peer review. As part of this commitment we make the peer review history of every article we publish publicly available.

When an article is published we post the peer reviewers' comments and the authors' responses online. We also post the versions of the paper that were used during peer review. These are the versions that the peer review comments apply to.

The versions of the paper that follow are the versions that were submitted during the peer review process. They are not the versions of record or the final published versions. They should not be cited or distributed as the published version of this manuscript.

BMJ Open is an open access journal and the full, final, typeset and author-corrected version of record of the manuscript is available on our site with no access controls, subscription charges or pay-per-view fees (http://bmjopen.bmj.com).

If you have any questions on BMJ Open's open peer review process please email info.bmjopen@bmj.com

BMJ Open

Study of Concussion in Rugby Union through MicroRNAs (SCRUM): A study protocol of a prospective, observational cohort study.

Journal:	*BMJ Open*
Manuscript ID	bmjopen-2018-024245
Article Type:	Protocol
Date Submitted by the Author:	18-May-2018

Complete List of Authors:	Yakoub, Kamal; NIHR Surgical Reconstruction and Microbiology Research Centre, University Hospitals Birmingham NHS Foundation Trust; Neurotrauma and Ophthalmology Research Group, Institute of Inflammation and Aging, University of Birmingham<br>O'Halloran, Patrick; Neurotrauma and Ophthalmology Research Group, Institute of Inflammation and Aging, University of Birmingham<br>Bentley, Conor; NIHR Surgical Reconstruction and Microbiology Research Centre, University Hospitals Birmingham NHS Foundation Trust<br>Davies, David; NIHR Surgical Reconstruction and Microbiology Research Centre, University Hospitals Birmingham NHS Foundation Trust; Neurotrauma and Ophthalmology Research Group, Institute of Inflammation and Aging, University of Birmingham<br>Forcione, Mario; NIHR Surgical Reconstruction and Microbiology Research Centre, University Hospitals Birmingham NHS Foundation Trust; Neurotrauma and Ophthalmology Research Group, Institute of Inflammation and Aging, University of Birmingham<br>Watson, Callum; Neurotrauma and Ophthalmology Research Group, Institute of Inflammation and Aging, University of Birmingham<br>Scarpa, Ugo; Neurotrauma and Ophthalmology Research Group, Institute of Inflammation and Aging, University of Birmingham<br>Bishop, Jon; NIHR Surgical Reconstruction and Microbiology Research Centre, Medical Statistician<br>Toman, Emma; Head Injury Management Research Group, Faculty of Clinical and Biomedical Science, School of Dentistry, University of Central Lancashire<br>Hammond, Douglas; Head Injury Management Research Group, Faculty of Clinical and Biomedical Science, School of Dentistry, University of Central Lancashire<br>Cross, MJ; Department for Health, University of Bath<br>Stokes, Keith; University of Bath, Department for Health<br>Kemp, Simon; Rugby Football Union, Twickenham<br>Di Pietro, V; NIHR Surgical Reconstruction and Microbiology Research Centre, University Hospitals Birmingham NHS Foundation Trust; Neurotrauma and Ophthalmology Research Group, Institute of Inflammation and Aging, University of Birmingham<br>belli, antonio; NIHR Surgical Reconstruction and Microbiology Research Centre, University Hospitals Birmingham NHS Foundation Trust; Neurotrauma and Ophthalmology Research Group, Institute of Inflammation and Aging, University of Birmingham

Keywords:	Sport concussion, traumatic brain injury, concussion, biomarkers, chronic traumatic encephalopathy, miRNA

Note: The following files were submitted by the author for peer review, but cannot be converted to PDF. You must view these files (e.g. movies) online.

RFU_Saliva_Urine_Sampling_V2.mp4

SCHOLARONE™ Manuscripts

For peer review only

Title

Study of Concussion in Rugby Union through MicroRNAs (SCRUM): A study protocol of a prospective, observational cohort study.

Authors

Kamal Makram Yakoub,[1,2] Patrick O'Halloran,[2] Conor Bentley,[1] David James Davies,[1,2] Mario Forcione,[1,2] Callum Nicholas Watson,[2] Ugo Scarpa,[2] Jonathan R B Bishop,[1] Emma Toman,[3] Douglas Hammond,[3] Matthew Joseph Cross,[5,6] Keith Stokes,[5] Simon PT Kemp,[6] Valentina Di Pietro,[1,2,4] Antonio Belli [1,2]

# Correspondence to

Dr Kamal Makram Yakoub

NIHR Surgical Reconstruction and Microbiology Research Centre

University Hospitals Birmingham NHS Foundation Trust

4th Floor West, Institute of Translational Medicine

Heritage Building (Queen Elizabeth Hospital)

B15 2TH

Birmingham

United Kingdom

E-mail: k.yakoub@bham.ac.uk

Telephone: +44(0)7460693705

Author affiliations

1 NIHR Surgical Reconstruction and Microbiology Research Centre, University Hospitals Birmingham NHS Foundation Trust, Birmingham, UK

2 Neurotrauma and Ophthalmology Research Group, Institute of Inflammation and Aging, University of Birmingham, Birmingham, UK

3 Head Injury Management Research Group, Faculty of Clinical and Biomedical Science, School of Dentistry, University of Central Lancashire, Preston, UK

4 The Beckman Institute for Advanced Science and Technology, University of Illinois at Urbana Champaign, Illinois, USA

5 Department for Health, University of Bath, Bath, UK

6 Rugby Football Union, Twickenham, London, UK

Word count 2169

Keywords

Sport concussion; traumatic brain injury; concussion; biomarkers; chronic traumatic encephalopathy; miRNA

Abstract

Introduction

The diagnosis of mild traumatic brain injury (mTBI) or sport related concussion (SRC) is a challenge for all clinicians, players, coaches and parents involved in contact sports. Currently, there is no validated objective biomarker available to assess the presence or severity of concussion in sport and so it is necessary to rely on subjective measures like self-reporting of symptoms which depend on the cooperation of the athlete. There is a significant health risk associated with repetitive injury if the diagnosis is missed and so there is great value in an objective biomarker to assist diagnostic and prognostic decisions.

Objective

To establish a panel of non-invasive MicroRNA biomarkers in urine and saliva for the rapid diagnosis of SRC and investigate the kinetics and clinical utility of these biomarkers in assisting diagnostic, prognostic and return-to-play decisions.

Methods and analysis

Observational, prospective, multicenter cohort study recruiting between the 2017-2018 rugby union season. Three groups of semi- and professional male rugby players will be recruited to the study: control athletes both uninjured and with musculoskeletal injuries and athletes who enter the World Rugby Head Injury Assessment (HIA) process during rugby matches in the Aviva Premiership and RFU Championship competitions. Saliva and urine will be collected from these athletes at multiple timepoints, in line with the HIA process.

For peer review only

Ethics and dissemination

The SCRUM study currently runs under University of Birmingham (UoB) ethics (ERN_11-0429AP28) as part of the Repetitive Concussion in Sport (RECOS) study.

This study was approved by the East of England - Essex Research Ethics Committee (REC) on 22 September 2017 – REC 17/EE/0275; IRAS 216703.

The complied and analysed results will be presented at national and international conferences concerning the care of traumatically brain injured patients. Results will also be submitted for peer review and publication in the subject journals/literature.

Article summary

Strengths and limitations of this study

-This study will contribute to a better understanding of the pathophysiology of sport related concussion and the role of MicroRNAs within this.

-The study will prospectively recruit elite rugby union athletes with potential and confirmed symptoms of SRC as well as elite athlete controls both with and without musculoskeletal trauma injuries.

-This work will potentially provide a novel means of noninvasively and objectively diagnosing SRC and advising on decisions relating to symptomatic treatment and return-to-play.

-The study will allow for the modelling of this novel diagnostic tool in relation to the existing widely used HIA process to understand the value which might be added by salivary and urinary markers.

For peer review only

-This study will recruit from a population of elite male athletes and so applications to other populations, such as female athletes, will require further study.

For peer review only - http://bmjopen.bmj.com/site/about/guidelines.xhtml

Introduction

Sport Related Concussion is form of mild traumatic brain injury caused by biomechanical forces transmitted directly or indirectly to the head and neck. Data from the Rugby Football Union (RFU) indicate that concussion occurs at an incidence of 15.8/1000 player hours in elite level rugby union in England .[1] It is a condition which, in the majority of cases results in a rapid onset but short-lived impairment of neurological function which usually resolves spontaneously.[2] It is accompanied by an absence of findings on conventional neuroimaging and, while 80-90% of sufferers are able to return to pre-injury levels within two week,[3] 20-30% go on to experience persistent post-concussion symptoms.[4]

Trends in the incidence of concussion over recent years appear to show rising rates, however it is felt that this likely reflects a rising awareness of the problem, rather than a true increase in rate and this mirrors a significant interest in this problem in the popular press generated in part by stories of catastrophic short term consequences and persistent disability following concussions or repeated concussions along with extensive litigation in the USA.[2, 3, 5, 6]

Despite the popular media attention surrounding concussion there remain a number of unanswered questions with regards to both the short and long-term consequences of this condition.[7] The relationship between concussive and subconcussive injuries cumulatively and later development of neurodegenerative disease such as Chronic Traumatic Encephalopathy (CTE), the potential for catastrophic injury (Second Impact Syndrome) following a premature return to head contact activities following concussion and the rehabilitation of patient suffering prolonged post concussive symptoms all require further exploration. The

For peer review only

issue safe return to sport following concussion is of particular note here in light of evidence of a prolonged period of metabolic disturbance which outlasts symptomatic recovery.[8]

In part, the difficulty in answering these questions fully relates to the challenge of diagnosing concussion itself, which continues to be an issue across all sports. It is recognised that the on-field assessment of players for signs and symptoms of concussion is challenging[9, 10] and that there is at present no perfect test with which to diagnose concussion.

In order to combat the current diagnostic conundrum, expert consensus recommends the use of multimodal diagnostic tools, at multiple timepoints to allow for the varied and evolving presentations of concussion to be picked up by clinicians.[9-13] However, the need for player engagement, the availability of clinicians and baseline information for athletes and the variation in diagnostic threshold between clinicians means that it is still challenging to establish a diagnosis and so to establish exposure to concussions in athletes.

One example of sport trying to facilitate the diagnosis of concussion is in the form of the World Rugby Head Injury Assessment (HIA) Process. This describes a series of clinical signs and symptoms which may be observed in real time or with the assistance of video replay. The presence of these features, if the diagnosis of concussion is not obvious, results in players being temporarily substituted and removed from the pitch in order to undergo a standardised 10-minute clinician evaluation.[11, 12]

For peer review only - http://bmjopen.bmj.com/site/about/guidelines.xhtml

Regardless of the outcome of this evaluation, the player is then reviewed again at the end of the match and at 36-48 hours to provide the multiple time point assessments to allow diagnosis of both early and late developing signs of concussion. This process is administered by the RFU in the English elite leagues and so represents an excellent opportunity to access players who have been diagnosed with concussion via a comprehensive and standardised diagnostic process.

Away from elite sport, there is, if anything, more variation in the diagnostic thresholds and safe return-to-activity advice which is given to patients and military personnel. This is reflected in the most recent UK National Institute for Health and Care Excellence (NICE) guidance which has highlighted the importance of TBI biomarker research in head injury.[14]

MicroRNAs (miRNA) are a recently discovered class of non-coding RNAs that play key roles in the regulation of gene expression. They are attracting increasing interest in clinical research as potential biomarkers for the identification and classification of cancers as well as other disease states including neurodegenerative diseases and most recently, brain trauma.[15, 16] This is reflected in the exponentially increasing number of publications on their use in a variety of medical applications.

Work done by DiPietro and colleagues (2017) has demonstrated the value of serum miRNA in providing prognostic information regarding the severity of traumatic brain injury in road traffic collision patients. Furthermore, this has been shown to be of value from very early timepoints, including at the roadside.[15] In light of the potential challenges of using an invasive serum test in an athletic setting, follow up work in the Birmingham Sport Concussion Clinic at the Queen Elizabeth Hospital

For peer review only

Birmingham (QEHB) has looked at the presence of miRNA markers in the blood, saliva and urine of concussed athletic patients presenting to the clinic within 72 hours of injury and healthy controls. This has identified 5 miRNA markers which are upregulated in saliva following concussion, demonstrating the potential promise of this category of fluid biomarkers in assisting with this diagnostic challenge (unpublished data).

Therefore, our objective is to establish a panel of non-invasive miRNA biomarkers in urine and saliva for the rapid diagnosis of sport-related concussion and investigate the kinetics and clinical utility of these biomarkers in assisting diagnostic, prognostic and return-to-play decisions.

Methods and analysis

Study design

The SCRUM study is a prospective observational study of all players (up to 1100) in the two highest tiers of domestic rugby competition in England, the Premiership and Championship. The study is embedded within the ongoing RFU Injury Surveillance Programme for the 2017-2018 season.

All players will be asked to provide approximately 200microL of saliva and about 5ml of urine during the preseason preparations at their clubs as well as completing a consent form detailing the processing and handling of their samples, the anonymisation of their data and their right to withdraw their voluntary consent at any time. These samples will provide the baseline miRNA values for a large population of elite athletes and are taken alongside a baseline questionnaire which provides information on previous concussion history and symptoms.

For peer review only

In addition to this baseline collection from the players, the club medical teams will be visited and provided with an explanation of the study materials and sample collection procedures. This will be reinforced with regular e-mail reminders during the season, the availability of the study team via telephone or e-mail 24/7 to answer questions and an online video demonstrating the collection procedure (see supplementary video).

During the season each time a player enters the HIA process (including if the player exhibits clear signs of concussion, for example loss of consciousness, categorized as an "Immediate Permanent Removal"), he will be asked to provide a saliva sample during the in match HIA. During the post-match and 36-48 HIA timepoints further saliva and urine samples are collected.

The HIAs at each time point take the form of a modified Sport Concussion Assessment Tool 5th Edition (SCAT5)[17] which has been well studied as a diagnostic tool in the context of sport related concussion and is complemented by baseline performance data in these assessments collected by the clubs during their preseason preparations.

In this way, we will be able to investigate the presence of concussion diagnostic miRNAs noninvasively at multiple timepoints which are matched to a standardised clinical assessment and diagnostic threshold for concussion performed by a clinician with experience in rugby head injuries.

In the case of players who are diagnosed with concussion at any time point, they will also be asked for a sample of saliva and urine at the point at which they are cleared by their team physician to return to rugby (Stage V) as per the Berlin

For peer review only

Consensus Graduated Return-to-Play (GRTP) protocol.[2] This will allow for the time course for recovery of the identified miRNA markers to be mapped.

Whenever a player is removed for an HIA, club medical teams will also be asked to approach an "uninjured control' player for saliva and urine samples after the game and at 36-48 hours later. An "uninjured control" is a non-concussed player who has played approximately a matched number of minutes in the same game. This will allow for the description of miRNA profiles in response to the physical stress of athletic activity and exposure to the likely subconcussive blows which come from participation in a collision sport.

To rule out contamination from non-neurological injuries and musculoskeletal trauma, club medics will also be asked to collect samples from players removed from the field of play due to musculoskeletal injuries (orthopaedic controls) at the same timepoints as the "uninjured control" group. The matching ratio for these groups (concussion, uninjured controls and orthopaedic controls) will be 1:1:1 (see table 1).

Finally, to study the potential effects on miRNA baseline profile of repeated involvement in a collision sport a cohort of clubs will be visited to invite athletes to provide a repeat urine and saliva samples during their preseason preparations in the 2018-2019 season.

For peer review only

Table 1. SCRUM sample collection schema

**CONCUSSED PLAYER**					
**Test**	Time point				
	Baseline	Pitch-side	Post-match	36h-48h post injury	Stage V GRTP
**HIA**		X	X	X	
**SALIVA**	X	X	X	X	X
**URINE**	X		X	X	X

**ORTHOPAEDIC CONTROL PLAYER**					
**Test**	Time point				
	Baseline	Pitch-side	Post-match	36h-48h post injury	Stage V GRTP
**HIA**					
**SALIVA**	X		X	X	
**URINE**	X		X	X	

**UNINJURED PLAYER**					
**Test**	Time point				
	Baseline	Pitch-side	Post-match	36h-48h post injury	Stage V GRTP
**HIA**					
**SALIVA**	X		X	X	
**URINE**	X		X	X	

For peer review only

The samples will be collected in Oragene®-RNA saliva collection pots and Norgen Biotech urine sample pots, both of which contain a proprietary miRNA stabilizing solution. The samples will be transported to the University of Birmingham (UoB) where they will be processed in line with the sample pot manufacturers guidance to allow freezing and storage.

A Next-generation sequencing (NGS) procedure will be carried out by the RNA sequencing services of Exiqon on an unbiased preliminary selection of samples in each of the three categories to allow identification of promising miRNA targets and these will then be characterized further in the whole population of study samples using quantitative PCR.

Sample size

The RFU estimates that out of the 1,100 players participating in the top two tiers of English rugby (The Premiership and Championship) each season, 350-400 are removed for a HIA, of whom approximately 200 will be diagnosed as concussed. If participation was universal, the study would generate approximately 3500 saliva samples and 2,900 urine samples from the following cohorts during the course of a season:

- 1,100 players tested preseason
- Approximately 200 players tested post-season
- 200 players with a definitive diagnosis of concussion following HIAs (tested at approximately four time points after injury)
- 150-200 players temporarily removed from play for suspected concussion but for whom concussion is not confirmed following HIAs (tested at three timepoints in concomitance with HIAs)
- 200 matched orthopaedic controls tested twice

For peer review only

- 200 matched uninjured post-game controls tested twice

Outcome measures and statistical analysis

Primary outcome(s)

The primary outcome measure will be the post-match levels of a panel miRNAs in a player's saliva sample following their initial removal for an HIA1.

Secondary outcomes

Secondary outcomes are miRNA levels in the saliva and urine samples at each stage following assessment until full recovery (e.g. at HIA2, HIA3 and at full recovery/end of stage V of the GRTP).

Timing of outcomes

The primary outcome assessment time is at initial removal for HIA.

The sample size estimations are based on a 2-sided ANOVA with four groups for pairwise comparisons (concussed, baseline, orthopaedic controls and uninjured controls), 90% power and 5% alpha.

All primary analyses (primary and secondary outcomes including safety outcomes) will made according to the matched groups (concussion, uninjured controls and orthopaedic controls) they were classified in at the time of the HIA. The primary comparison will be between concussed players and uninjured post-game controls.

Biochemical and imaging data will also be analysed with standard group comparison statistics (e.g. t-test on normalised data or Mann-Whitney on non-normalisable data).

For peer review only

Patient and Public Involvement

The Rugby Players' Association (RPA) was involved from the outset and members were asked to share their views on the design and setting up of this study. They are also part of the steering and oversight group. In terms of dissemination, we have kept clubs updated on the progress of the study and will report the findings by email/newsletter and in various meetings where representatives are invited.

Ethics and dissemination

This study has been approved by the East of England - Essex Research Ethics Committee on 22 September 2017 – REC 17/EE/0275; IRAS 216703.

The study is funded by the University of Birmingham (UoB) and the National Institute for Health Research Surgical Reconstruction and Microbiology Research Centre (NIHR SRMRC) for 3 years.

All data will be collected and kept on NHS servers in accordance with the 1998 UK Data Protection Act, UoB and QEHB data handling and maintenance guidelines, with the minimum amount of required information recorded. The computers on this network have restricted physical access; data are stored under coded filenames and the local network has secure password access restricted to a limited set of people.

All the complied and analysed results will be presented at national and international conferences concerning the care of the traumatically brain injured patient. Results will also be submitted for peer review and publication in the subject journals/literature.

The datasets generated during and/or analysed during the current study are/will be available upon request from, Professor Antonio Belli, who is the point of contact

For peer review only

and will be able to provide anonymised data with sufficient detail to able to reproduce the analyses for up to 10 years.

Acknowledgements

The authors acknowledge UoB and NIHR SRMRC for their support. Also, we would like to thank the RFU, RPA and all the rugby clubs that agreed to participate in the study and for their valuable feedback on the design and setting up of the study.

Author Contributions

AB is the principal investigator.
AB, VD, SPTK, MJC, JRB developed the protocol.
AB, CB, JRB, DH, MJC, SPTK, VD, KS drafted the protocol and commented on the protocol.
KMY, DJD, CB, MF, CNW, POH, US, ET, VD drafted and finalised the manuscript.
All authors have approved the final manuscript.

Funding

This work is supported by the University of Birmingham (UoB) grant number ERN_11-0429AP28.

Competing interests

None declared.

Patient consent

Not required.

Ethics approval

This study has been approved by the East of England - Essex Research Ethics Committee (REC) on 22 September 2017 – REC 17/EE/0275; IRAS 216703.

For peer review only

References

1 Rugby Football Union. The professional rugby injury surveillance project: the 2015-16 annual report. 2017 http://www.englandrugby.com/mm/Document/General/General/01/32/25/17/1516_PRISP_Annual_Report_FINAL%28withcontentspage%29_English.pdf (accessed 16 May 2018).

2 Brett BL, Kuhn AW, Yengo-Kahn AM, et al. On-field signs predict future acute symptoms after sport-related concussion: a structural equation modeling study. Journal of the International Neuropsychological Society 2018;1-0.

3 Broglio SP, Collins MW, Williams RM, et al. Current and emerging rehabilitation for concussion: a review of the evidence. Clinics in sports medicine 2015;34(2):213-31.

4 Heitger MH, Jones RD, Macleod AD, et al. Impaired eye movements in post-concussion syndrome indicate suboptimal brain function beyond the influence of depression, malingering or intellectual ability. Brain 2009;132(10):2850-70.

5 Toman E, Harrisson S, Belli T. Biomarkers in traumatic brain injury: a review. Journal of the Royal Army Medical Corps 2016;162:103-108.

6 Cross M, Kemp S, Smith A, et al. Professional Rugby Union players have a 60% greater risk of time loss injury after concussion: a 2-season prospective study of clinical outcomes. Br J Sports Med 2016;50:926-931.

7 Kuhn AW, Yengo-Kahn AM, Kerr ZY, et al. Sports concussion research, chronic traumatic encephalopathy and the media: repairing the disconnect. Br J Sports Med 2017;51:1732-1733.

8 Kamins J, Bigler E, Covassin T, et al. What is the physiological time to recovery after concussion? A systematic review. Br J Sports Med 2017;51:935-940.

9 McCrory P, Meeuwisse WH, Echemendia RJ, et al. What is the lowest threshold to make a diagnosis of concussion?. Br J Sports Med 2013;47:268-271.

10 Putukian M, Raftery M, Guskiewicz K, et al. Onfield assessment of concussion in the adult athlete. Br J Sports Med 2013;47:285-288.

11 Fuller GW, Kemp SPT, Decq P. The International Rugby Board (IRB) Pitch Side Concussion Assessment trial: a pilot test accuracy study. Br J Sports Med 2015;49:529-535.

12 Fuller CW, Fuller GW, Kemp SPT, et al. Evaluation of World Rugby's concussion management process: results from Rugby World Cup 2015. Br J Sports Med 2017;51:64-69.

13 Patricios J, Fuller GW, Ellenbogen R, et al. What are the critical elements of sideline screening that can be used to establish the diagnosis of concussion? A systematic review. Br J Sports Med 2017;51:888-894.

14 The National Institute for Health and Care Excellence (NICE). Head injury: assessment and early management. 2017 https://www.nice.org.uk/guidance/cg176 (accessed 16 May 2018).

15 Di Pietro V, Ragusa M, Davies D, et al. MicroRNAs as novel biomarkers for the diagnosis and prognosis of mild and severe traumatic brain injury. Journal of neurotrauma. 2017;34(11):1948-56.

16 Redell JB, Moore AN, Ward III NH, et al. Human traumatic brain injury alters plasma microRNA levels. Journal of neurotrauma. 2010;27(12):2147-56.

17 Echemendia RJ, Meeuwisse W, McCrory P, et al. The Sport Concussion Assessment Tool 5th Edition (SCAT5): Background and rationale. Br J Sports Med 2017;51:848-850.

For peer review only

BMJ Open

Study of Concussion in Rugby Union through MicroRNAs (SCRUM): A study protocol of a prospective, observational cohort study.

Journal:	*BMJ Open*
Manuscript ID	bmjopen-2018-024245.R1
Article Type:	Protocol
Date Submitted by the Author:	16-Aug-2018

Complete List of Authors:	Yakoub, Kamal; NIHR Surgical Reconstruction and Microbiology Research Centre, University Hospitals Birmingham NHS Foundation Trust; Neurotrauma and Ophthalmology Research Group, Institute of Inflammation and Aging, University of Birmingham<br>O'Halloran, Patrick; Neurotrauma and Ophthalmology Research Group, Institute of Inflammation and Aging, University of Birmingham<br>Davies, David; NIHR Surgical Reconstruction and Microbiology Research Centre, University Hospitals Birmingham NHS Foundation Trust; Neurotrauma and Ophthalmology Research Group, Institute of Inflammation and Aging, University of Birmingham<br>Bentley, Conor; NIHR Surgical Reconstruction and Microbiology Research Centre, University Hospitals Birmingham NHS Foundation Trust<br>Watson, Callum; Neurotrauma and Ophthalmology Research Group, Institute of Inflammation and Aging, University of Birmingham<br>Forcione, Mario; NIHR Surgical Reconstruction and Microbiology Research Centre, University Hospitals Birmingham NHS Foundation Trust; Neurotrauma and Ophthalmology Research Group, Institute of Inflammation and Aging, University of Birmingham<br>Scarpa, Ugo; Neurotrauma and Ophthalmology Research Group, Institute of Inflammation and Aging, University of Birmingham<br>Bishop, Jon; NIHR Surgical Reconstruction and Microbiology Research Centre, Medical Statistician<br>Toman, Emma; Head Injury Management Research Group, Faculty of Clinical and Biomedical Science, School of Dentistry, University of Central Lancashire<br>Hammond, Douglas; Head Injury Management Research Group, Faculty of Clinical and Biomedical Science, School of Dentistry, University of Central Lancashire<br>Cross, MJ; Department for Health, University of Bath<br>Stokes, Keith; University of Bath, Department for Health<br>Kemp, Simon; Rugby Football Union, Twickenham<br>Menon, David; NIHR Global Health Research Group for Neurotrauma, University of Cambridge<br>Di Pietro, V; NIHR Surgical Reconstruction and Microbiology Research Centre, University Hospitals Birmingham NHS Foundation Trust; Neurotrauma and Ophthalmology Research Group, Institute of Inflammation and Aging, University of Birmingham<br>belli, antonio; NIHR Surgical Reconstruction and Microbiology Research Centre, University Hospitals Birmingham NHS Foundation Trust;

	Neurotrauma and Ophthalmology Research Group, Institute of Inflammation and Aging, University of Birmingham
**Primary Subject Heading**:	Sports and exercise medicine
Secondary Subject Heading:	Sports and exercise medicine, Neurology, Diagnostics
Keywords:	Sport concussion, traumatic brain injury, concussion, biomarkers, chronic traumatic encephalopathy, miRNA
Note: The following files were submitted by the author for peer review, but cannot be converted to PDF. You must view these files (e.g. movies) online.	
RFU_Saliva_Urine_Sampling_V2.mp4	

SCHOLARONE™
Manuscripts

Title

Study of Concussion in Rugby Union through MicroRNAs (SCRUM): A study protocol of a prospective, observational cohort study.

Authors

Kamal M Yakoub,[1,2] Patrick O'Halloran,[2] David J Davies,[1,2] Conor Bentley,[1] Callum N Watson,[2] Mario Forcione,[1,2] Ugo Scarpa,[2] Jonathan RB Bishop,[1] Emma Toman,[3] Douglas Hammond,[3] Matthew J Cross,[4,5] Keith A Stokes,[4,6] Simon PT Kemp,[6] David K Menon,[7] Valentina Di Pietro,[1,2,8] Antonio Belli [1,2]

# Correspondence to

Dr Kamal Makram Yakoub

NIHR Surgical Reconstruction and Microbiology Research Centre

University Hospitals Birmingham NHS Foundation Trust

4th Floor West, Institute of Translational Medicine

Heritage Building (Queen Elizabeth Hospital)

B15 2TH

Birmingham

United Kingdom

E-mail: k.yakoub@bham.ac.uk

Telephone: +44(0)7460693705

Author affiliations

1 NIHR Surgical Reconstruction and Microbiology Research Centre, University Hospitals Birmingham NHS Foundation Trust, Birmingham, UK

2 Neurotrauma and Ophthalmology Research Group, Institute of Inflammation and Aging, University of Birmingham, Birmingham, UK

3 Head Injury Management Research Group, Faculty of Clinical and Biomedical Science, School of Dentistry, University of Central Lancashire, Preston, UK

4 Department for Health, University of Bath, Bath, UK

5 Premiership Rugby, Twickenham, London, UK

6 Rugby Football Union, Twickenham, London, UK

7 Division of Anaesthesia, Addenbrooke's Hospital, University of Cambridge, Cambridge, UK

8 The Beckman Institute for Advanced Science and Technology, University of Illinois at Urbana Champaign, Illinois, USA

Keywords

Sport concussion; traumatic brain injury; concussion; biomarkers; chronic traumatic encephalopathy; miRNA

Abstract

Introduction

The diagnosis of mild traumatic brain injury (mTBI) or sports-related concussion is a challenge for all clinicians, players, coaches and parents involved in contact sports. Currently, there is no validated objective biomarker available to assess the presence or severity of concussion in sport and so it is necessary to rely on subjective measures like self-reporting of symptoms which depend on the cooperation of the athlete. There is a significant health risk associated with repetitive injury if the diagnosis is missed and so there is great value in an objective biomarker to assist diagnostic and prognostic decisions.

Objective

To establish a panel of non-invasive MicroRNA biomarkers in urine and saliva for the rapid diagnosis of sports-related concussion and investigate the kinetics and clinical utility of these biomarkers in assisting diagnostic, prognostic and return-to-play decisions.

Methods and analysis

Observational, prospective, multicenter cohort study recruiting between the 2017-2018 and 2018-2019 rugby union seasons. Professional rugby players in the two highest tiers of senior professional domestic rugby competition in England will be recruited prospectively to the study. During the season three groups will be identified: athletes entering the World Rugby Head Injury Assessment (HIA) protocol, uninjured control athletes and control athletes with musculoskeletal injuries saliva and urine will be collected from these athletes at multiple

For peer review only

timepoints, coinciding with key times in the HIA protocol and return-to-play process.

Ethics and dissemination

Ethics approval has been obtained. The compiled and analysed results will be presented at national and international conferences concerning the care of traumatically brain injured patients. Results will also be submitted for peer review and publication in the subject journals/literature.

Article summary

Strengths and limitations of this study

-This study will contribute to a better understanding of the pathophysiology of sports-related concussion and the role of MicroRNAs within this.

-The study will prospectively recruit elite rugby union athletes entering the World Rugby Head Injury Assessment (HIA) protocol, before their diagnosis of sports-related concussion is confirmed, as well as elite athlete controls both with and without musculoskeletal trauma injuries.

-This work will potentially provide a novel means of noninvasively and objectively diagnosing sports-related concussion and advising on decisions relating to symptomatic treatment and return-to-play.

-The study will allow for the modelling of this novel diagnostic tool in relation to an existing, robust and widely used multimodal concussion assessment process (the World Rugby HIA protocol) to understand the value which might be added by salivary and urinary markers.

For peer review only

-This study will recruit from a population of elite male adult athletes and so applications to other populations, such as female and youth athletes, will require further study.

For peer

Introduction

Sports-related concussion is a form of mild traumatic brain injury caused by biomechanical forces transmitted directly or indirectly to the head and neck. Data from the Rugby Football Union (RFU) indicate that concussion occurs at an incidence of 20.9/1000 player hours in elite level rugby union in England.**[1]** In the majority of cases, concussion results in a rapid onset, but short-lived, impairment of neurological function that resolves spontaneously.**[2]** It is accompanied by an absence of findings on conventional neuroimaging and, while 80-90% of the sufferers are able to return to pre-injury levels of neurocognitive and physical functions within two weeks, 2.5% go on to experience persistent post-concussion symptoms (e.g. headaches, dizziness, fatigue, memory problems).**[3,4]**

Concussion incidence has increased in rugby union over recent years,**[1]** however it is felt that this, at least in part, reflects a rising awareness of the problem, rather than a true increase in rate. This mirrors a significant interest in this injury in the popular press generated in part by stories of catastrophic short term consequences and persistent disability following concussions or repeated concussions along with extensive litigation in the USA.**[2, 3, 5]**

Despite the popular media attention surrounding concussion there remain a number of unanswered questions with regards to both the short and long-term consequences of this condition.**[6]** The relationship between the cumulative burden of concussive and sub-concussive injuries and significant clinical outcomes remains uncertain. These outcomes include the later development of neurodegenerative disease such as Chronic Traumatic Encephalopathy (CTE), the potential for catastrophic injury (Second Impact Syndrome) following a premature

For peer review only

return to head contact activities following concussion, and the need for rehabilitation of patient suffering prolonged post concussive symptoms; all of which require further exploration. The issue of safe return to sport following concussion is of particular note here in light of evidence of a prolonged period of metabolic disturbance**[7]** and increased risk of subsequent injuries when returning to play,**[8]** both of which outlast symptomatic recovery.

In part, the difficulty in answering these questions fully relates to the challenge of diagnosing concussion itself, which continues to be an issue across all sports. It is recognised that the on-field assessment of players for signs and symptoms of concussion is challenging**[9, 10]** and that there is at present no perfect test with which to diagnose concussion.

In order to combat current diagnostic uncertainty, expert consensus recommends the use of multimodal diagnostic tools, at multiple timepoints to allow for the varied and evolving presentations of concussion to be picked up by clinicians.**[9-13]** However, the need for player engagement, the availability of clinicians, the need for baseline athlete information, and the variation in diagnostic threshold between clinicians means that it is still challenging to establish a definitive diagnosis of sports-related concussion in athletes.

One example of sport attempting to facilitate diagnosis of concussion is the Head Injury Assessment (HIA) protocol in elite level Rugby Union. This protocol has been described previously and has been evaluated and shown to reduce the proportion of players being allowed to continue playing after suffering a concussion.**[11, 12]**

For peer review only

Briefly, players who demonstrate clear observable signs or symptoms of concussion (defined as conforming to any one of 11 "Criteria 1" signs or symptoms) following a head injury event either observed directly by medical staff or following video review or confirmed during an on pitch medical assessment (e.g. loss of consciousness, tonic posturing, nystagmus) are permanently removed from the game without any further off field concussion screening and, irrespective of further clinical evaluation, are considered to have sustained a concussion.

Where the outcome of a head impact event is not clear, for example if no "Criteria 1" signs or symptoms are identified but there is a suspicion that the injury mechanism may have resulted in a concussion (defined as conforming to a set of "Criteria 2" signs), the players undergo a standardised off-field assessment. This off field screening assessment takes the form of the multi-modal "HIA1" assessment involving Maddock's questions, symptom checklist, immediate and delayed recall, tandem gait test and assessment of clinical signs. A window of ten minutes is permitted for this assessment to be conducted with the player being temporarily replaced in the game for that period. Any abnormality in the HIA1 assessment and/or a clinical suspicion of concussion by the assessing practitioner mandates a permanent removal from play, but a diagnosis of concussion is not made or excluded at this time point. An electronic decision aid is used to assist this assessment with reference to baseline data collected in preseason for each player.

All players who undergo the HIA1 assessment, irrespective of outcome, have more detailed clinical assessments post-match (HIA2) and again after two nights rest (HIA3) to monitor their progress and confirm or exclude a diagnosis of concussion. The HIA2 assessment involves the use of the Sport Concussion Assessment tool 5th edition (SCAT-5),**[14]** tandem gait test and clinical assessment, while the HIA3

For peer review only

may also include the use of neurocognitive testing (typically computerised tools such as Cogsport).

The diagnosis of concussion then is reached either during the game on the basis of the presence of standardised "Criteria 1" features, or in the 48 hours following the match on the basis of two subsequent clinical assessments by the team physician at the HIA2 and HIA3 time-points, supported by the standardised HIA2 or HIA3 assessments conducted by the team physician.

Therefore this process divides players into three groups:

- Players who are immediately and permanently removed from the game on the basis of "Criteria 1" features (Immediate Permanent Removal, IPR) and diagnosed with concussion.
- Players who enter the HIA1 screening process and are not diagnosed with concussion following the completion of either of their HIA2 and HIA3 assessments.
- Players who enter the HIA1 screening process and are diagnosed with concussion at either their HIA2 or HIA3 assessments.

Away from elite sport, there is, if anything, more variation in the diagnostic thresholds and safe return-to-activity advice which is given to patients and military personnel. This is reflected in the most recent UK National Institute for Health and Care Excellence (NICE) guidance which has highlighted the importance of TBI biomarker research in head injury.**[15]**

MicroRNAs (miRNA) are a recently discovered class of non-coding RNAs that play key roles in the regulation of gene expression. They are attracting increasing interest in clinical research as potential biomarkers for the identification and

For peer review only

classification of cancers as well as other disease states including neurodegenerative diseases and most recently, brain trauma.**[16, 17, 18]** This is reflected in the exponentially increasing number of publications on their use in a variety of medical applications.

Work by DiPietro and colleagues (2017) has demonstrated the value of serum miRNA in providing prognostic information regarding the severity of traumatic brain injury in road traffic collision patients. Furthermore, this has been shown to be of value from very early timepoints, including at the roadside.**[16]** In light of the potential challenges of using an invasive serum test in an athletic setting, follow up work in the Birmingham Sport Concussion Clinic at the Queen Elizabeth Hospital Birmingham (QEHB) has looked at the presence of miRNA markers in the blood, saliva and urine of concussed athletic patients presenting to the clinic within 72 hours of injury and healthy controls. This has identified five miRNA markers which are upregulated in saliva following concussion, demonstrating the potential promise of this category of fluid biomarkers in assisting with this diagnostic challenge.**[19]** Therefore, our objective is to establish a panel of non-invasive miRNA biomarkers in urine and saliva for the rapid diagnosis of sports-related concussion and investigate the kinetics and clinical utility of these biomarkers in assisting diagnostic, prognostic and return-to-play decisions.

For peer review only

Methods and analysis

Study design

The SCRUM study is a prospective observational cohort study of all male players (up to 1100) in the two highest tiers of senior professional domestic rugby competition in England, the Premiership and Championship. The study is

embedded within the ongoing RFU's Professional Rugby Injury Surveillance Programme for the 2017-2018 and 2018-2019 seasons.

All players will be visited at their club during the pre-season period and provided with information about the study, including the processing and handling of their samples, the anonymisation of their data and their right to withdraw their voluntary consent at any time, before being asked to complete a consent form. They will then be asked to provide a single baseline sample of approximately 2ml of saliva and about 5ml of urine, if possible before their usual training session. These samples will provide the baseline miRNA values for a large population of elite athletes and are taken alongside a baseline questionnaire which provides information on previous concussion history and symptoms.

In addition to this baseline collection from the players, the club medical teams will be visited and provided with an explanation of the study materials and sample collection procedures. This will be reinforced with regular e-mail reminders during the season, the availability of the study team via telephone or e-mail 24/7 to answer questions, and an online video demonstrating the collection procedure (see supplementary video).

During the season, each time a player enters the HIA process he will be asked for saliva sample at the HIA1 timepoint and for saliva and urine samples at the HIA2 and HIA3 timepoints. In this way, we will be able to investigate the presence of concussion diagnostic miRNAs noninvasively at multiple timepoints which are matched to a standardised clinical assessment and diagnostic threshold for concussion performed by a clinician with experience in rugby head injuries.**[12]**

For peer review only

Players diagnosed with concussion will be asked for a sample of saliva and urine at the point at which they are cleared by their team physician to return to rugby (Stage V) as per the Berlin Consensus Graduated Return-to-Play (GRTP) protocol.**[20]** This will allow for the time course for recovery of the identified miRNA markers to be mapped. Data regarding the identification of players entering the HIA process and returning to play are collected weekly by the RFU and Premiership Rugby which will allow us to monitor compliance through the season and to analyse differences between incidents included and not included in our data collection.

Whenever a player enters the HIA process, club medical teams will also be asked to approach an "uninjured control" player for saliva and urine samples at the HIA2 and HIA3 timepoints. An "uninjured control" is a non-concussed player who has played approximately a matched number of minutes in the same game. This will allow for the description of miRNA profiles in response to the physical stress of athletic activity and exposure to the likely sub-concussive blows which come from participation in a collision sport.

To rule out contamination from non-neurological injuries and musculoskeletal trauma, club medics will also be asked to collect samples from players removed from the field of play due to musculoskeletal injuries (orthopaedic controls) at the same timepoints as the "uninjured control" group. The matching ratio for these groups (concussion, uninjured controls and orthopaedic controls) will be 1:1:1 (see table 1).

For peer review only

Table 1. SCRUM sample collection schema

**CONCUSSED PLAYER**					
**Test**	Time point				
	Baseline	Pitch-side	Post-match	36h-48h post injury	Stage V GRTP
**HIA**	X	X (HIA1)	X (HIA2)	X (HIA3)	
**SALIVA**	X	X	X	X	X
**URINE**	X		X	X	X

**ORTHOPAEDIC CONTROL PLAYER**					
**Test**	Time point				
	Baseline	Pitch-side	Post-match	36h-48h post injury	Stage V GRTP
**HIA**	X				
**SALIVA**	X		X	X	
**URINE**	X		X	X	

**UNINJURED PLAYER**					
**Test**	Time point				
	Baseline	Pitch-side	Post-match	36h-48h post injury	Stage V GRTP
**HIA**	X				
**SALIVA**	X		X	X	
**URINE**	X		X	X	

For peer review only

The samples will be collected in Oragene®-RNA saliva collection pots and Norgen Biotech urine sample pots, both of which contain a proprietary miRNA stabilizing solution. The samples will be transported to the University of Birmingham (UoB) where they will be processed in line with the sample manufacturers guidance to allow freezing and storage.

A Next-generation sequencing (NGS) procedure will be carried out by the RNA sequencing services of Exiqon on an unbiased preliminary selection of an estimated 20 samples in each of the different players groups. NGS allows the identification of all potential non-coding RNAs that are differentially expressed between study groups. The NGS analysis will potentially identify a number of suitable candidate biomarkers. To correct for the effect of multiple comparisons, we will apply the Benjamini-Hochberg procedure to select biomarkers with a false discovery rate < 0.05 for further analysis by quantitative PCR in the whole population of study samples. From the surviving panel, we will exclude biomarkers whose concentrations are not significantly different from matched baseline values (p value > 0.05).

Sample size

The RFU estimates that out of the 1100 players participating in the top two tiers of English rugby (The Premiership and Championship) each season, 350-400 players enter the HIA process, of whom approximately 200 will be diagnosed as concussed. If participation was universal, the study would generate approximately 3500 saliva samples and 2900 urine samples from the following cohorts during the course of a season:

- 1100 players tested preseason.

For peer review only

- Approximately 200 players will provide repeat baseline samples in the post-season. This will allow for quality control for intra-individual variation from season to season.
- 200 players with a definitive diagnosis of concussion following either identification of "Criteria 1" signs in-game or following the HIA2 and HIA3 assessments in the 48 hours post-game.
- 150-200 players temporarily removed from play for an off field assessment following a head injury event (possible concussion) but for whom concussion is not subsequently confirmed following the HIA2 and HIA3 assessments in the 48 hours post-game.
- 200 matched controls with musculoskeletal injuries tested twice.
- 200 matched uninjured controls tested twice.

Outcome measures and statistical analysis

Biomarker data will be analysed with standard group comparison statistics (e.g. t-test on normalised data or Mann-Whitney on non-normalisable data).

Primary outcome(s)

The primary outcome measure will be the post-match (HIA2) comparison of levels of a panel of miRNAs in saliva and urine samples from players who have been definitively diagnosed with concussion through the HIA process described above vs players in the uninjured and musculoskeletal injury control groups described above.

For peer review only

Secondary outcomes

Secondary outcomes are:

- Kinetics of the sample biomarkers at the different timepoints outlined in the protocol above in Table1.
- We will also report HIA1, 2 and 3 levels of the same miRNA panel for players entering the HIA process who were not eventually diagnosed with concussion.
- Correlation between levels of specific biomarkers and severity, type and duration of symptoms as well as time to return to full participation.

For peer review only

Patient and Public Involvement

The Rugby Players' Association (RPA) is the representative body for professional players in England and was involved from the outset, with members asked to share their views on the design and setting up of this study. They are also part of the steering and oversight group. In terms of dissemination, we will keep clubs updated on the progress of the study and will report the findings by email/newsletter and in various meetings where club representatives are in attendance.

Ethics and dissemination

The SCRUM study runs under University of Birmingham (UoB) ethics (ERN_11-0429AP28) as part of the Repetitive Concussion in Sport (RECOS) study.

This study was also approved by the East of England - Essex Research Ethics Committee (REC) on 22 September 2017 – REC 17/EE/0275; IRAS 216703. This study will also utilise data collected by the RFU for injury surveillance, our use of which has been approved by University of Bath Research Ethics Approval Committee for Health (EP 16/17 286).

The study is funded by the Medical Research Council (MRC), National Institute for Health Research (NIHR), Biovici Diagnostics Ltd and the Rugby Football Union (RFU), grant number: MRC CIC 164132, for 3 years.

All data will be collected and kept on NHS servers in accordance with the 1998 UK Data Protection Act, UoB and QEHB data handling and maintenance guidelines, with the minimum amount of required information recorded. The computers on this network have restricted physical access; data are stored under coded filenames and the local network has secure password access restricted to a limited set of people.

All the compiled and analysed results will be presented at national and international conferences concerning the care of the traumatically brain injured patient. Results will also be submitted for peer review and publication in the subject journals/literature.

The datasets generated during and/or analysed during the current study are/will be available upon request from, Professor Antonio Belli, who is the point of contact and will be able to provide anonymised data with sufficient details to reproduce the analyses for up to 10 years.

For peer review only

Acknowledgements

The authors acknowledge UoB and NIHR SRMRC for their support. Also, we would like to thank the RFU, RPA and all the rugby clubs that agreed to participate in the study for their valuable feedback on the design and setting up of the study.

Author Contributions

AB, VDP are the principal investigators.
AB, VDP, SPTK, MJC, JRB developed the protocol.
AB, JRB, DH, MJC, SPTK, DKM, VDP, KS, DJD drafted the protocol and commented on the protocol.
KMY, POH, DJD, CB, MF, CNW, US, ET, VDP drafted and finalised the manuscript.
All authors have approved the final manuscript.

Funding

This work is supported by funding received from the Medical Research Council (MRC), National Institute for Health Research (NIHR), Biovici Diagnostics Ltd and the Rugby Football Union (RFU), grant number: MRC CIC 164132.

Competing interests

The University of Birmingham owns intellectual property for some of the biomarkers used in this study.

Patient consent

Not required.

Ethics approval

The SCRUM study runs under University of Birmingham (UoB) ethics (ERN_11-0429AP28) as part of the Repetitive Concussion in Sport (RECOS) study. This study was also approved by the East of England - Essex Research Ethics Committee (REC) on 22 September 2017 – REC 17/EE/0275; IRAS 216703. This study will also utilise data collected by the RFU for injury surveillance, our use of which has been approved by University of Bath Research Ethics Approval Committee for Health (EP 16/17 286).

For peer review only

References

1 Rugby Football Union. The professional rugby injury surveillance project: the 2016-17 annual report. 2017 https://www.englandrugby.com/mm/Document/General/General/01/32/91/95/InjurySurveillanceReport2016-17_English.pdf (accessed 30 July 2018).

2 Brett BL, Kuhn AW, Yengo-Kahn AM, et al. On-field signs predict future acute symptoms after sport-related concussion: a structural equation modeling study. Journal of the International Neuropsychological Society 2018;1-0.

3 Broglio SP, Collins MW, Williams RM, et al. Current and emerging rehabilitation for concussion: a review of the evidence. Clinics in sports medicine 2015;34(2):213-31.

4 Heitger MH, Jones RD, Macleod AD, et al. Impaired eye movements in post-concussion syndrome indicate suboptimal brain function beyond the influence of depression, malingering or intellectual ability. Brain 2009;132(10):2850-70.

5 Toman E, Harrisson S, Belli T. Biomarkers in traumatic brain injury: a review. Journal of the Royal Army Medical Corps 2016;162:103-108.

6 Kuhn AW, Yengo-Kahn AM, Kerr ZY, et al. Sports concussion research, chronic traumatic encephalopathy and the media: repairing the disconnect. Br J Sports Med 2017;51:1732-1733.

7 Kamins J, Bigler E, Covassin T, et al. What is the physiological time to recovery after concussion? A systematic review. Br J Sports Med 2017;51:935-940.

8 Cross M, Kemp S, Smith A, et al. Professional Rugby Union players have a 60% greater risk of time loss injury after concussion: a 2-season prospective study of clinical outcomes. Br J Sports Med 2016;50:926-931.

9 McCrory P, Meeuwisse WH, Echemendia RJ, et al. What is the lowest threshold to make a diagnosis of concussion?. Br J Sports Med 2013;47:268-271.

10 Putukian M, Raftery M, Guskiewicz K, et al. Onfield assessment of concussion in the adult athlete. Br J Sports Med 2013;47:285-288.

11 Fuller GW, Kemp SPT, Decq P. The International Rugby Board (IRB) Pitch Side Concussion Assessment trial: a pilot test accuracy study. Br J Sports Med 2015;49:529-535.

12 Fuller CW, Fuller GW, Kemp SPT, et al. Evaluation of World Rugby's concussion management process: results from Rugby World Cup 2015. Br J Sports Med 2017;51:64-69.

13 Patricios J, Fuller GW, Ellenbogen R, et al. What are the critical elements of sideline screening that can be used to establish the diagnosis of concussion? A systematic review. Br J Sports Med 2017;51:888-894.

14 Echemendia RJ, Meeuwisse W, McCrory P, et al. The Sport Concussion Assessment Tool 5th Edition (SCAT5): Background and rationale. Br J Sports Med 2017;51:848-850.

15 The National Institute for Health and Care Excellence (NICE). Head injury: assessment and early management. 2017 https://www.nice.org.uk/guidance/cg176 (accessed 16 May 2018).

16 Di Pietro V, Ragusa M, Davies D, et al. MicroRNAs as novel biomarkers for the diagnosis and prognosis of mild and severe traumatic brain injury. Journal of neurotrauma. 2017;34(11):1948-56.

17 Redell JB, Moore AN, Ward III NH, et al. Human traumatic brain injury alters plasma microRNA levels. Journal of neurotrauma. 2010;27(12):2147-56.

18 Di Pietro V, Yakoub KM, Scarpa U, et al. MicroRNA Signature of Traumatic Brain Injury: From the Biomarker Discovery to the Point-of-Care. Frontiers in Neurology. 2018;9.

19 Di Pietro V, Porto E, Ragusa M, et al. Salivary microRNAs: diagnostic markers of mild traumatic brain injury in contact-sport. Frontiers in Molecular Neuroscience. 2018;11:290.

20 McCrory P, Meeuwisse W, Dvorak J, et al. Consensus statement on concussion in sport—the 5th international conference on concussion in sport held in Berlin, October 2016. Br J Sports Med 2017;51:838-847.

iew only
